# Genetic and cellular characterization of MscS-like putative channels in the filamentous fungus *Aspergillus nidulans*

**DOI:** 10.1080/19336950.2022.2098661

**Published:** 2022-08-08

**Authors:** Mariangela Dionysopoulou, Nana Yan, Bolin Wang, Christos Pliotas, George Diallinas

**Affiliations:** a Astbury Centre for Structural Molecular Biology, School of Biomedical Sciences, University of Leeds, LS2 9JT, Leeds, United Kingdom; b Department of Biology, National and Kapodistrian University of Athens, Panepistimioupolis, 15784 Athens, Greece; c Institute of Molecular Biology and Biotechnology, Foundation for Research and Technology, 70013 Heraklion, Greece

**Keywords:** Mechanosensitive, ion channels, MscS, fungi, AlphaFold

## Abstract

Mechanosensitive ion channels are integral membrane proteins ubiquitously present in bacteria, archaea, and eukarya. They act as molecular sensors of mechanical stress to serve vital functions such as touch, hearing, osmotic pressure, proprioception and balance, while their malfunction is often associated with pathologies. Amongst them, the structurally distinct MscL and MscS channels from bacteria are the most extensively studied. MscS-like channels have been found in plants and *Schizosaccharomyces pombe*, where they regulate intracellular Ca^2+^ and cell volume under hypo-osmotic conditions. Here we characterize two MscS-like putative channels, named MscA and MscB, from the model filamentous fungus *Aspergillus nidulans*. Orthologues of MscA and MscB are present in most fungi, including relative plant and animal pathogens. MscA/MscB and other fungal MscS-like proteins share the three transmembrane helices and the extended C-terminal cytosolic domain that form the structural fingerprint of MscS-like channels with at least three additional transmembrane segments than *Escherichia coli* MscS. We show that MscA and MscB localize in Endoplasmic Reticulum and the Plasma Membrane, respectively, whereas their overexpression leads to increased CaCl_2_ toxicity or/and reduction of asexual spore formation. Our findings contribute to understanding the role of MscS-like channels in filamentous fungi and relative pathogens.

## Introduction

Mechanosensitive (MS) ion channels are present in the membranes of organisms across all three domains of life, bacteria, archaea, and eukarya. They are the molecular sensors for a diverse range of functions including touch, hearing, proprioception, balance, and osmotic homeostasis [1–4]. MS channels have been first identified in *Escherichia coli* and form two structurally distinctive families, MscL [5–8] and MscS [9–15]. MscL and MscS although evolutionary and structurally distinct, share functional properties such as sensitivity to membrane tension. They offer protection against osmotic shock to different extents, by acting as pressure safety valves. The six identified *E. coli* MscS-like channels present a large variety in their sizes, number and topology of their transmembrane (TM) segments and functional properties [16–19]. Despite progress on the smaller *E. coli* MscS-like family members, characterization of the large ones, namely YbiO, MscK and MscM is currently lacking [16, 20, 21]. MscS-like channels exist in most bacterial species, plants and protozoa, but not in metazoa, while in plants they are involved in defense mechanisms against bacterial pathogens or they increase the infectivity of the latter to eukaryotic hosts [22–25]. Plant MscS-like homologues are involved in osmotic stress, touch, vibration, and also allow plants to distinguish up from down by sensing the force of gravity [26–30]. All MscS-like channels possess a similar TM network of three helices which forms inner-leaflet pockets that are implicated in the mechanosensitivity of MscS [3, 9, 11, 31, 32]. Similar pockets have been also identified in the structurally distinct MscL channel and shown to influence its mechanical gating properties [7, 33–35]. These pockets have first been first characterized as a unique structural feature on MscS by Electron Paramagnetic Resonance (EPR) spectroscopic methods [10, 12, 36] and later confirmed by X-ray crystallography and cryo-electron microscopy (cryo-EM), which in most cases have enabled pocket lipids to be resolved [9, 15, 26, 28, 37]. These findings led to the entropy driven lipid-moves first model according to which lipid occupancy within the pockets determines the state of MscS [3, 9, 11, 31, 32], a model derived from the force-from-lipid principle [38]. Subtle sequence differences within the pockets were shown to lead to different channel architectures which could account for functional differences between homologous MscL [34, 39], where binding of compounds within these MscL pockets inhibits cell growth and alters MscL’s gating properties. MscS-like channels vary in selectivity for the permeating ions from being nonselective between anions and cations in bacteria, to presenting cation selectivity, which allows passage Ca^2+,^ K^+^ and Na^+^ in eukaryotes.

In fungi, *Schizosaccharomyces pombe* Msy1 and Msy2 MscS-like channels localize at the Endoplasmic Reticulum (ER) and seem to regulate intracellular Ca^2+^ and cell volume for survival upon hypo-osmotic conditions [40]. Here we present genetic and cellular characterization of two *Aspergillus nidulans* MscS-like putative channels, named MscA and MscB. Orthologues of MscA and MscB are present in all filamentous fungi, but not in *Saccharomyces cerevisiae* and most *Saccharomycetes*. We characterize MscA and MscB null and overexpression mutants relative to colony growth and cell morphology under various physiological or stress conditions. We further study the steady-state subcellular sorting of the GFP-tagged MscA and MscB channels and show that MscA and MscB localize in the ER and plasma membrane (PM), respectively. Finally, we generate AlphaFold models and show that MscA and MscB have related but distinctive topologies to other MscS-like channels in plants, fungi and bacteria. Our findings pave the way for elucidating the physiological role and distinct functional properties of MscS-like channels in filamentous fungi and relative pathogens.

### Identification of two new putative MscS-like channels in Aspergillus nidulans

We used the fission yeast MscS homologues, Msy1 and Msy2, as *in silico* probes to identify *via* blastp searches homologous proteins of *A. nidulans* in FungiDB (https://fungidb.org/fungidb). Two proteins showed significant scores, namely the products of genes annotated as AN7571 and AN6053. Both genes/proteins are predicted to encode “proteins that have a role in cellular volume homeostasis, calcium ion homeostasis, regulation of resting membrane potential and the cortical ER localization” (https://www.uniprot.org/). We thereafter named the proteins corresponding to AN7571 and AN6053 as MscA and MscB, respectively. MscA has 37.4% and 29.3% amino acid identity with Msy1 and Msy2, while MscB has 29.9% and 32.7% amino acid identity with Msy1 and Msy2. While the two *S. pombe* proteins have a significant difference in length per subunit (1011 *versus* 840 amino acids), due to longer N- and C-termini in Msy1, the *A. nidulans* proteins are of similar size (MscA: 952 and MscB: 944, residues), but still differ significantly in the length of their N- and C-termini (MscA has a 50 amino acids longer N-tail, while MscB has a 36 amino acids longer C-tail). A primary sequence alignment (**Figure S1**) shows that MscA is significantly more similar to Msy1 than Msy2, whereas MscB is more similar to Msy2 than Msy1. The fungal proteins share significant similarity (> 22–23% identity) with the *E. coli* MscS channel not only in the TM1 (α2), TM2 (α3) and TM3 (α4) helices (residues ~20-130), but also in the extended cytosolic domain (~130-280), that together constitute the structural fingerprint of MscS-like channels. Notably, the cytosolic part the fungal proteins and MscS share a highly conserved “signature” motif, namely PNX2ΦΧ4Φ2ΧNX2R, in the β4-α5-β5 sheets (**Figure S1**). The fungal proteins overall share high sequence similarity between themselves and along their full length, except for their terminal regions.

**Figure 1.**
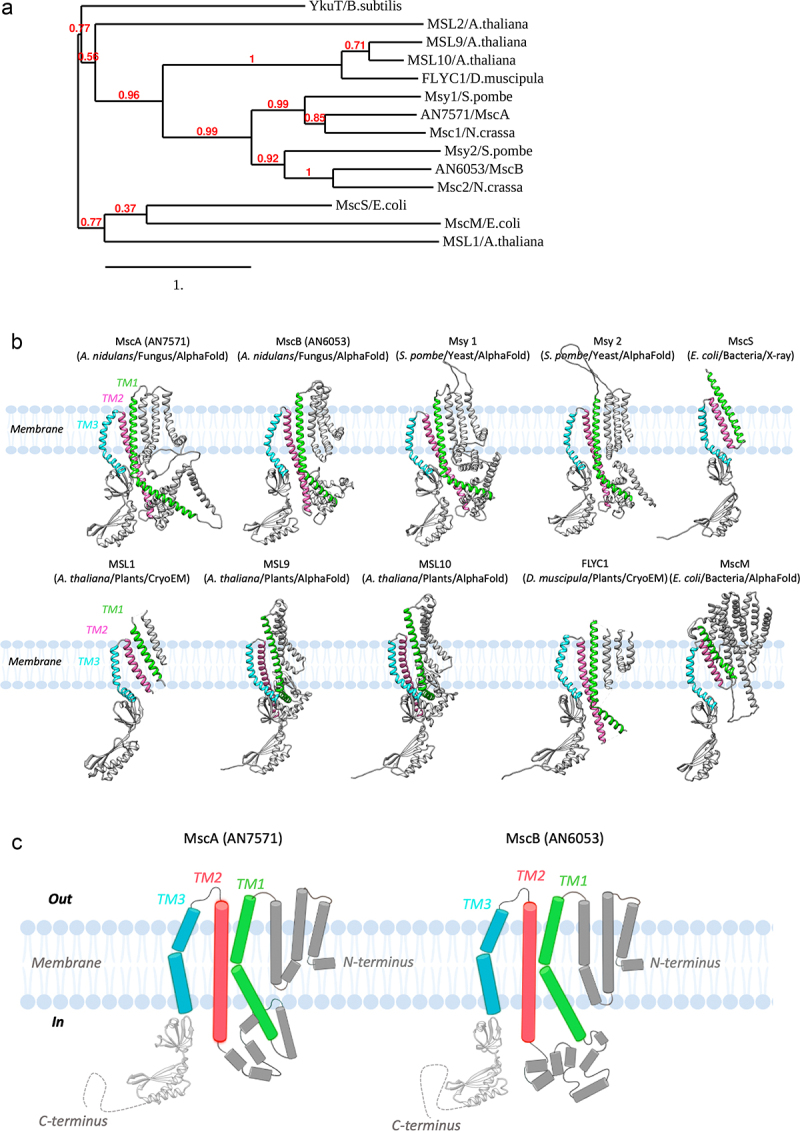
***In silico* analysis and AlphaFold modeling of MscA and MscB. A**. Phylogenetic analysis of MscA and MscB. The selected MscS-like proteins are: MscS and MscM of *E. coli*, YkuT of *B. subtilis*, MscA and MscB of *A. nidulans*, Msy1 and Msy2 of *S. pombe*, MSL1, MSL2, MSL9, MSL10 of *A. thaliana*, and FLYC1 from the plant flycatcher (*D. muscipula*). MSL9 and MSL10 are functionally characterized plasma membrane channels, whereas, MSL1 is an organellar (inner mitochondrial membrane) MscS-like channel protein [51]. Notice the distinct bacterial/plant organellar, fungal and plant clades, and the relationship of MscA and MscB with Msy1 and Msy2 of *S. pombe*, respectively. Values closer to 1 suggest a better separation between the species inthe phylogenetic tree. The tree was made using http://www.phylogeny.fr/. **B**. AlphaFold prediction modeling of MscA and MscB single subunits and representative MscS-like structural models from fungi, plants and bacteria. MscA and MscB from *A. nidulans*, Msy1 and Msy2 from *S. pombe*, MscS (PDB: 5AJI) and MscM from *E. coli*, MSL1 (PDB: 6VXM), MSL9 and MSL10 from *A. thaliana*, and FLYC1 from the plant flycatcher (PDB: 7N5D). Notice that the predicted N-terminal and C-terminal residues with low confidence (<70 pLDDT) for MscA, MscB, Msy1 and Msy2 are not included. *E. coli* MscS was used for structural alignment using Chimera. TM1, TM2 and TM3 helices, which are the signature fold of the MscS-like channels and form the characteristic lipid pockets evident in all these channels are depicted in green, pink and cyan respectively. **C**. Topology prediction for MscA and MscB. Cartoon representation of MscA and MscB AlphaFold topology models generated with BioRender. The predicted TM1-3 and the following cytoplasm-facing domains constitute the signature sequence of MscS-like channels. The orientation of the N-terminal end in respect to whether it is facing the extracellular or intracellular space remains elusive.

Our phylogenetic analysis further supports that MscA and MscB are orthologues of Msy1 and Msy2, respectively. The tree shown in Figure 1A further suggests that fungal proteins group in a clade distinct from the bacterial (MscS, MscM and YkuT) and plant organellar homologues (MSL1 and MSL2), and are more related to a plant clade that includes MSL9 and MSL10 channel proteins known to function as PM MS channels in *Arabidopsis thaliana* [24, 27, 41], and the *Dionaea muscipula* (Venus flytrap) FLYC1 channel [28].

Fungal MscS homologues are found in all higher filamentous fungi (dikarya) and several primitive fungi (e.g. Glomeromycota or Entomophthoromycota), with similarities following evolutionary distances (e.g. 55–85% identity among *Aspergillus* homologues, 31–34% between *ascomycetes* and *basidiomycetes*, or 30–35% between *Aspergilli* and true yeasts, like *Yarrowia*). A notable evolutionary loss of MscS-like proteins is in the *Saccharomyces* group. All *Aspergilli* and most filamentous *ascomycetes* conserve close orthologues of MscA/AN7571 and McsB/AN6053 (> 70% identity).

Noticeably, MscS homologues have significantly different lengths. For example, *E. coli* MscS consists of 286 amino acids, plant organellar proteins are close to 500 amino acids, PM plant homologues are 734–742 amino acids, and fungal homologues are longer (840–1011 residues). This is reflected to 3–7 TM segments (*E. coli* MscS possesses 3 TM helices) and possibly a different orientation (extracellular *versus* intracellular) of their terminal ends.

To compare the structural architectures between single subunits among the *A. nidulans, S. pombe* and other MscS-like homologues we generated new AlphaFold models for MscA and MscB [42, 43]. In particular, we generated models for the *A. nidulans* MscA and MscB, and compared with AlphaFold models of Msy1 and Msy2 from *S. pombe*, MSL9 and MSL10 from *A. thaliana*, MscM from *E. coli*, as well as with experimentally determined by cryo-EM or X-ray crystallography structures of MSL1 from *A. thaliana*, FLYC1 from *D. muscipula* and MscS from *E. coli* [13, 26, 28] (Figure 1B). This also allowed us to validate the accuracy of the single subunit AlphaFold predictions for this family of proteins (Figure 1B). The N-terminus of *E. coli* MscS has been resolved in more recent CryoEM studies in nanodiscs, suggesting the lipid bilayer is important for its stability [15, 37]. However, in most cases, N-terminal AlphaFold predictions yielded low confidence scores (<70 pLDDT) and were excluded from further analysis. Therefore, we only included domains with residues which presented high AlphaFold prediction scores (>70 pLDDT) to generate the MscA and MscB models (**Figure S2**).

MscA and MscB possess the three characteristic TM1, TM2 and TM3 helices, which constitute the structural fingerprint of *E. coli* MscS, that form the lipid pockets, but they also include additional TM helices (i.e. 3–8), which may account for curvature sensitivity, as seen in orthologous channels [3, 9, 11, 28, 31, 32] (Figure 1B). The *A. nidulans* MscA/MscB proteins present a similar topology and TM architecture to the *S. pombe* homologues, (Figure 1B and 1C). Noticeably, however, the AlphaFold cannot confidently predict the orientation of the N-terminal end of the fungal MscS-like proteins in respect to their extracellular or intracellular localization.

### MscA and MscB are not essential for growth, but their overexpression leads to growth defects and increased Ca^2+^ toxicity

**Figure 2.**
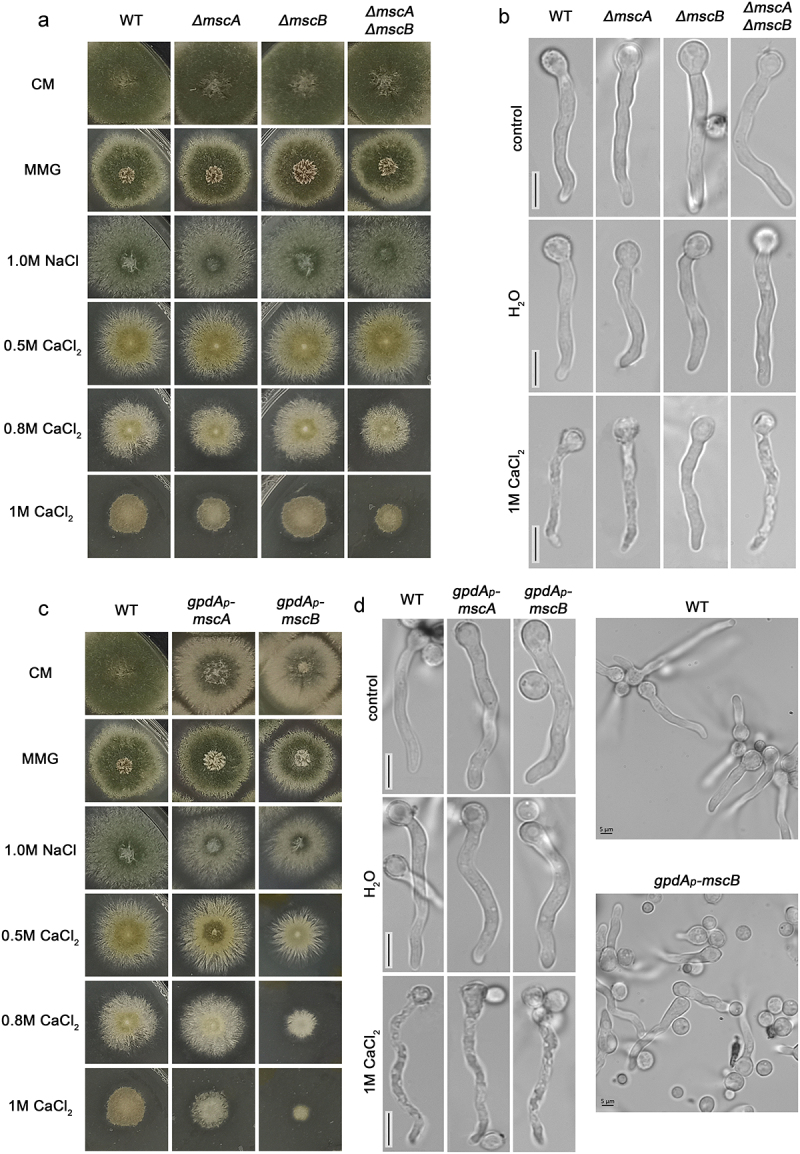
**Growth phenotypes and germling morphology of strains carrying null mutations or alleles overexpressing MscA or MscB. A**. Growth analysis of *knock-out* mutants Δ*mscA*, Δ*mscB* and Δ*mscA*/Δ*mscB* compared to an isogenic wild-type control strain on CM and MMG, and MMG containing 1.0 M NaCl or of 0.5 M-1.0 M CaCl_2_ at 37 ℃. Notice that growth rate and morphology of the *knock-out* strains are similar to those of the control strain in all conditions tested, with the exception of a mild increase inCaCl_2_ sensitivity of Δ*mscA* and Δ*mscA*/Δ*mscB* mutants. **B**. Microscopic morphology of germlings of *knock-out* strains grown for 16 h in MMG, or for 16 h in MMG followed by 1 h shift to hypotonic (H_2_O) or hypertonic (1 M CaCl_2_) conditions. Notice the reduced toxicity of CaCl_2_ in Δ*mscB*. **C**. Growth phenotypes of strains overexpressing MscA and MscB under the strong *gpdA* promoter, relative to isogenic control. Details are as in **A**. Notice that overexpressed MscB leads to reduced colony diameter, more evident in the presence of CaCl_2_ (1 M). **D**. Microscopic morphology of germling of strains overexpressing MscA and MscB compared to a control. Details are as in **B**. Hypotonic and hypertonic conditions did not seem to have a significant effect on germling morphology. Notice also, in the right panels, that the *mscB* overexpressing strain (bottom image) has significantly reduced germination, reflected in a very high number of non-germinated spores, contrasting the wt control strain where all spores have germinated in germling (upper image). Scale bars: 5 μm.

We constructed single and double null mutants of MscA and/or MscB using standard reverse genetic methodology based on protoplast transformation and homologous recombination events of linear DNA cassettes (see Materials and methods). The single mutants were constructed individually, and then were used as templates to construct the double mutant by genetic crossing. Deletion of the entire open reading frame of both genes was confirmed by PCR. The null mutants were tested in respect to their colony growth rate and morphology. Figure 2A shows that all null mutants (Δ*mscA*, Δ*mscB* and Δ*mscA*/Δ*mscB*) are viable and able to grow similar to an isogenic wild-type control on complete or minimal glucose media (CM and MMG) at pH 6.8 and 37 ℃. A similar picture was obtained in MMG containing various carbon or nitrogen sources, pH 5.5 or 8.0, and at 25 ℃ (not shown). We did not detect any growth modification of the null mutants in hyperosmotic media relative to the wild-type control (1.0 M NaCl). On the other hand, we detected, a very moderate increase in sensitivity to CaCl_2_ (0.8–1.0 M) of Δ*mscA*, confirmed also in Δ*mscA*/Δ*mscB*. This is reflected in 23–38% reduction in colony diameter (Figure 2A**, bottom rows)**. We did not detect any significant effect caused by the genetic absence of Δ*mscA* and /or Δ*mscB* in respect to the morphology and rate of germination of germlings under normal or hypotonic conditions (Figure 2B). The presence of CaCl_2_ (1.0 M) leads to apparent cell toxicity in wild-type control cells, evident as shrinkage and invagination of the PM and arrest of growth. The same effect was observed in Δ*mscA*, and Δ*mscA*/Δ*mscB*, but not in Δ*mscB*. Thus, MscB seems to contribute to CaCl_2_ toxicity, in line with growth tests (i.e. increased diameter of Δ*mscB* relative to wild-type and the Δ*mscA* mutant, Figure 2A), but also with the localization of MscB in the PM, as shown later in this work.

We further constructed and tested strains overexpressing MscA or MscB *via* the strong constitutive *gpdA*_p_ promoter [44]. Figure 2C shows that overexpression of MscB, but not MscA, leads to moderate reduced (~15%) colony diameter in CM, MMG and hypertonic media, and increased sensitivity to CaCl_2_ (i.e. >60% reduction in colony diameter). In addition, overexpression of both MscA and MscB led to reduction of conidiospore formation in CM. Figure 2D further shows that in control media the overexpression of MscA and MscB led to reduced conidiospore germination, growth delay and moderate swelling of germlings. In hypotonic media (H_2_O) we did not detect any additional morphological change, whereas in CaCl_2_ (1.0 M) both MscA and MscB overexpression led to faster vacuolarization and apparent germling death.

### MscA and MscB are localized in the ER and the PM respectively

**Figure 3.**
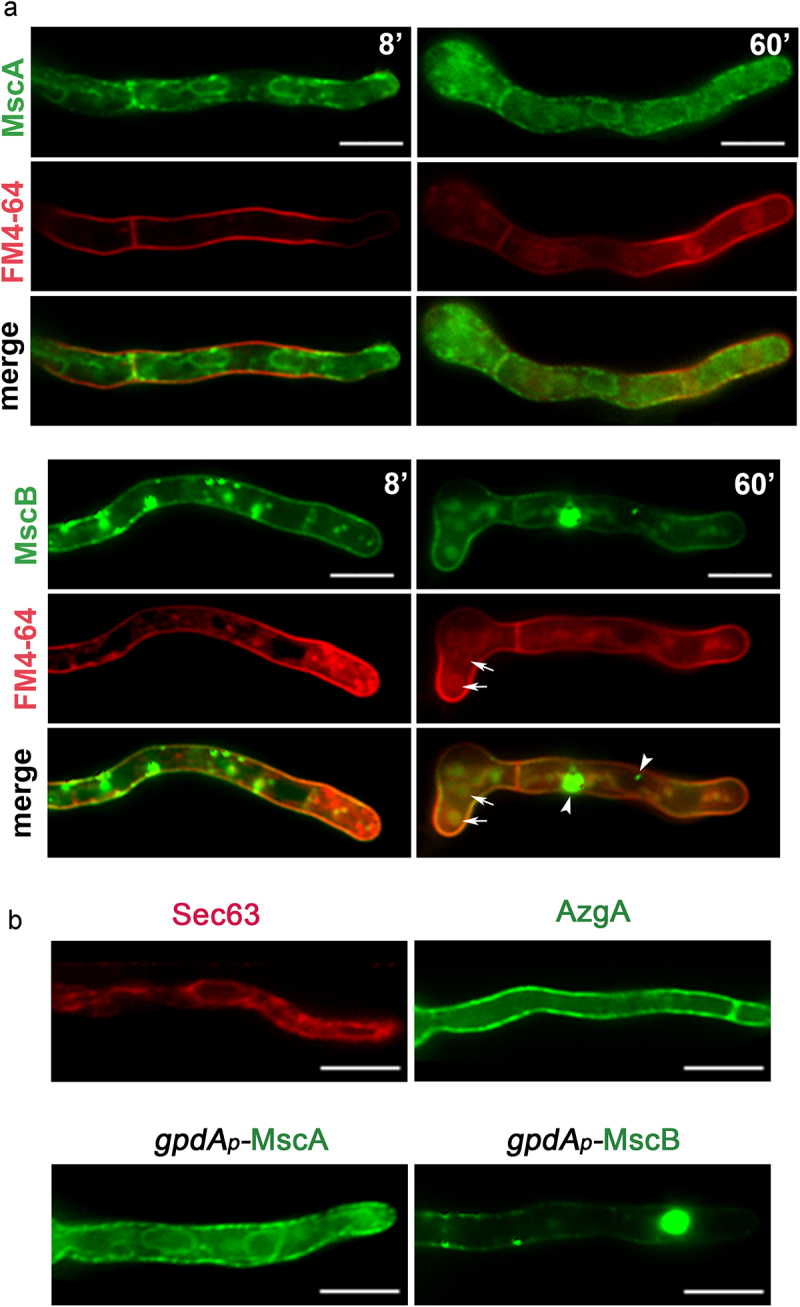
**Subcellular localization of MscA and MscB. A**. Epifluorescence microscopy of strains overexpressing GFP-tagged versions of MscA and MscB, co-stained with FM4-64. MscA labels the cortical and perinuclear ER network, but not the PM (upper panel). In the case of MscB significant co-localization with FM4-64 was obtained at the PM (lower panel). GFP-MscB also labels cytosolic aggregates (arrowheads) and vacuoles (arrows), as shown in 60 min pictures. **B**. Epifluorescence microscopy of GFP-tagged MscA and MscB compared to mCherry-Sec63 and AzgA-GFP, which are cortical ER and PM markers, respectively. Notice the rather similar localization of MscA and MscB with mCherry-Sec63 and AzgA-GFP, respectively. Scale bars: 5 μm.

In order to localize the subcellular compartment where MscA and MscB function, we have constructed by standard reverse genetics strains that express the two channels tagged N-terminally with eGFP. GFP-MscA and GFP-MscB chimeras were expressed from a standard moderate/strong constitutive promoter, namely *gpdA_p_* [44]. Figure 3A shows the epifluorescence microscopic analysis of GFP-MscA and GFP-MscB in growing germlings, also stained with the amphiphilic styryl dye FM4-64. FM4-64 rapidly stained the plasma membrane (see left panels after 8 min incubation) and was then dynamically integrated into endosomes and vacuoles (right panels, 60 min). GFP-MscA labeled the entire ER network, as reflected by the appearance of the fluorescent signal in perinuclear rings and an extended membranous network typical of cortical ER (notice the cortical ER signal just beneath the PM labeled with FM4-64). The non-continuous presence of the fluorescent signal associated with the PM at the septa confirms that MscA is localized in the cortical ER rather than the PM. The picture of homogeneous ER-localization of MscA is not compatible with ER-retention due to partial misfolding that is caused by the GFP tag, because misfolded TM proteins tend to accumulate in the perinuclear ER, rather than reaching the cortical ER. Thus, MscA should be a native ER protein in *A. nidulans*.

Distinctly from the ER localization of GFP-MscA, GFP-MscB labeled homogeneously the PM (notice the colorization with FM4-64). MscB also appeared in a few cytosolic aggregates and small vacuoles. The presence of GFP-MscB signal in vacuoles is normal, as TM proteins such as transporters and receptors are turned-over in this compartment, following a steady-state endocytic route. The presence of MscS-GFP in a small number of aggregates might be explained by the fact that we used a heterologous promoter for expression, which may lead to increased production of MscB. This however does not dismiss that GFP-MscB finds its path to the PM. In Figure 3B, we also compare the localization of MscA and MscB with AzgA and Sec63, markers of the PM and the cortical ER, respectively, further supporting that MscA is an ER protein, while MscB is PM-associated.

Our results are in partial disagreement with what has been reported for the *S. pombe* MscS-like orthologues [40]. More specifically, Msy1 and Msy2 have been reported to localize mainly to the endoplasmic reticulum, the former labeling exclusively perinuclear ER rings and the latter mostly on the periphery of cells, considered by the authors to correspond to the cortical ER. This was well-supported by the observation that Msy1 co-localized with BiP, a marker labeling mostly the perinuclear ER, whereas Msy2 co-localized with calnexin, a marker labeling mostly the cortical ER. However, in their subcellular fractionation experiment, after sucrose density gradient centrifugation, both Msy1 and Msy2 proteins seemed to co-fractionate mostly with Pma1 (proton efflux ATPase), a standard PM molecular marker, and only secondarily with BiP, a cortical ER marker. In addition, in [40] tagging of Msy1 and Msy2 was different to the one reported here for MscA and MscB. Msy1 was tagged with an HA epitope, rather than a fluorescent protein, which allowed its detection through a fluorescent anti-HA antibody in fixed rather than living cells. Msy2 was tagged N-terminally with mCherry, rather than GFP. Given the orthology of the *A. nidulans* and *S. pombe* MscS-like proteins and the fact that specific epitope tagging may cause partial misfolding and mislocalization of integral TM proteins, the lack of PM localization of Msy2 and the restricted presence of Msy2 in perinuclear rings in *S. pombe* may represent mislocalized fractions of these proteins, rather than reflecting real differences between the fungi.

## Materials and methods

### Media, strains, growth conditions and transformation

Standard complete and minimal media for *Aspergillus nidulans* were used (FGSC, http://www.fgsc.net). Media and chemical reagents were obtained from Sigma-Aldrich (Life Science Chemilab SA, Hellas) or AppliChem (Bioline Scientific SA, Hellas). Glucose 1% (w/v) was used as carbon source. NaNO_3_ was used as nitrogen sources at 10 mM. *A. nidulans* transformation was performed by generating protoplasts from germinating conidiospores using TNO2A7 as a recipient strain that allow selection of transformants via complementation of a pyrimidine autotrophy [45]. Integrations of gene fusions with fluorescent tags, promoter replacement fusions or deletion cassettes were selected using the *A. fumigatus* markers orotidine-5-phosphate decarboxylase (AF*pyrG*, Afu2g0836), GTP-cyclohydrolase II (AF*riboB*, Afu1g13300) or the panthogenic acid *pantoB100* resulting in complementation of the relevant auxotrophies. Transformants were verified by PCR analysis.

### Nucleic acid manipulations and plasmid constructions

Genomic DNA extraction from *A. nidulans* was performed as described in FGSC (http://www.fgsc.net). Plasmids, prepared in *Escherichia coli* (strain DH5a), and DNA restriction or PCR fragments were purified from agarose 1% (w/v) gels with the Nucleospin Plasmid Kit or Nucleospin ExtractII kit, according to the manufacturer’s instructions (Macherey–Nagel, Lab Supplies Scientific SA, Hellas). Standard PCR reactions were performed using KAPATaq DNA polymerase (Kapa Biosystems). PCR products used for cloning and re-introduction by transformation in *A. nidulans* were amplified by a high-fidelity KAPA HiFi HotStart Ready Mix (Kapa Biosystems) polymerase. pGEM-T-easy vector was used as template to amplify the relevant linear cassettes carrying an auxotrophic marker for *knock-out* strains by PCR. The ORFs of MscA and MscB were tagged with GFP at the N-terminus and inserted into the *SpeI/NotI* site of a modified pGEM-T-easy vector carrying a version of the *gpdA* promoter, the *trpC* 39 termination region, and the *panB* selection marker [46].

### Fluorescence microscopy

Conidiospores were incubated overnight in glass bottom 35 mm l-dishes (ibidi, Lab Supplies Scientific SA, Hellas) in liquid minimal media, for 16–20 h at 25°C, [1% (w/v) glucose] and then shifted to various conditions [dH_2_O or 1 M CaCl_2_] for 1–2 h. FM4-64 (Thermo Fischer Scientific, Waltham, MA, USA) staining was according to [47]. Images were obtained using an inverted Zeiss Axio Observer Z1 equipped with an Axio Cam HR R3 camera. Contrast adjustment, area selection and color combining were made using the Zenlite 2012 software. Scale bars were added using the FigureJ plugin of the ImageJ software. Images were further processed and annotated in Adobe Photoshop CS4 Extended version 11.0.2.

### Phylogenetic analysis and multiple sequence alignment

Phylogenetic analysis of Msc-like proteins was performed by http://www.phylogeny.fr/ [48]. Multiple sequence alignment was performed by http://multalin.toulouse.inra.fr/multalin/ [49].

### AlphaFold prediction and modeling

AlphaFold modeling was performed using Alphafold Colab prediction tools (https://colab.research.google.com/github/deepmind/alphafold/blob/main/notebooks/AlphaFold.ipynb) [42, 43]. Protein sequences for AN7571 and AN6053 and were obtained from FungiDB (https://fungidb.org/). Structure prediction was attempted for both sequences with 1–780 amino acids, as longer amino acids length attempts failed during server processing. *S. pompe* Msy1 and Msy2, *A. thaliana* MSL9 and MSL10 and *E. coli* MscM monomers were previously predicted by AlphaFold, and the PBD files were downloaded from the AlphaFold Protein Structure Database (https://alphafold.ebi.ac.uk/). Previously determined cryo-EM structures of MSL1 (PDB: 6VXM) [26], FLYC1 (PDB: 7N5D) [28], and an X-ray structure of *E. coli* MscS (PDB: 5AJI) [9] were downloaded from the Protein Database Bank (https://www.rcsb.org/). For all the predicted structures, residues with low confidence scores (<70 pLDDT) were not included in our analysis and figure illustrations. The monomer of the *E.coli* MscS was used as model for structural alignment for the nine proteins included in Figure 1B and figure was made using Chimera [50].

## Supplementary Material

Supplemental Material

## Data Availability

The authors confirm that the data supporting the findings of this study are available within the article [and/or] its supplementary materials.
