# Supplementary material for: Genetic and cellular characterization of MscS-like putative channels in the filamentous fungus Aspergillus nidulans
Source: Channels (Austin). 2022 Aug 8;16(1):148–58. doi: 10.1080/19336950.2022.2098661 (PMC9367656; doi:10.1080/19336950.2022.2098661)
Supplement: Supplemental Material [file KCHL_A_2098661_SM7602.pdf]

MscS/E.coli

```
MscS/E.coli   ............................................................
Msy1/S.pombe  MSSPTSPTSSPGRHHWSNSKDGMPPEYTNQDPNSDQADNENSDAKAHQPQHSPQHSTENQ
AN7571/MscA   ..........................................MTTPVNEVPEPSYPAAER
AN6053/MscB   ............................................................
Msy2/S.pombe  ............................................................
```

MscS/E.coli

```
MscS/E.coli   ............................................................
Msy1/S.pombe  GHTGTSDTSSLEMELSKLHPESKQRQLPHSPEHERSRSPIASVVSYRSHMTLEDENQIFN
AN7571/MscA   RRRSDSDSSPTVDGNAYVHDESQ....PHDGAYAGAQ........YGSHLQVDTTFQDFD
AN6053/MscB   .............................................MSGRPGLAEKRLSSH
Msy2/S.pombe  ............................................................
```

MscS/E.coli

```
MscS/E.coli   ............................................................
Msy1/S.pombe  AEMAVRGSQSLQRRPTGRSVRGSMRRLSSHRSKSMRTSKSKKSGDYERTAENEEAAQEAE
AN7571/MscA   KTSRSPSAAREQAMRLEDDLAVLEAERVASRS.THGTEKDARSGDVHSLTRSRSRRAEDV
AN6053/MscB   RFQQIPDTMPNPNDVTIDIPLTSVSSRGQTGGYSAGAEHNG.GAEKGGLTSSPPSSSLGF
Msy2/S.pombe  ..................................MNEHRREPHRRSGYQDDSAFTNTEKL
```

MscS/E.coli

```
MscS/E.coli   ............................................................
Msy1/S.pombe  NHLDNFGVVTFGTEAPIKAPDHPVTIFGRIFKFIQQRSFYLRSLIYIIPLGVLLLIPVFI
AN7571/MscA   DEFDEATNPLHEKAAVYNPPENPSTGVSKFIKRVHESSFIVRYFTYIVPLVLILLIPLLV
AN6053/MscB   GHRRRRTINDKTGLPAEEPEDGTVTRMGRFYQAVLNFSTVTRYLIYIAPLAALLAIPIIV
Msy2/S.pombe  VDELDHNVEPEQLLEKNRTDFKLMYVIVKFYRWFNNLSFITRWITIWFPLAGALVIPLAV
```

MscS/E.coli

```
MscS/E.coli   ............................................................
Msy1/S.pombe  GRFYHPQPPYRDELGHEYERHLHVGGVDLMWMAIWWEIIWLSIWAARYAAKVIPYFFAFF
AN7571/MscA   GALAYPDAS..............VGGVELLWFSVWLEIVWLTLWAGRIVAKLLPTPVNIF
AN6053/MscB   GATAAEDAK..............IGGVSLPWFFCWVEVVWVSLWVCKLVAKVIPFVFQFV
Msy2/S.pombe  GVSPYPNAK..............LGGVRIFWIFVWLEVAWGGFWVSRVIARLLPYILYPL
```

MscS/E.coli

```
MscS/E.coli   ............................................................
Msy1/S.pombe  VSFISNNVTKWRCMAVALEFPITLFLWMLAVYVSFLPIMT....................
AN7571/MscA   ASIFTNNSKKWRDVAKQLELPVTLFLWWLGVEISFLPTMK....................
AN6053/MscB   CGIVSAGTRKYALILRNLEIPITMVLWMIVSLVTFLPIMV....................
Msy2/S.pombe  MGILPFTMYKYTVILTALEMPLAIFFCSIVCVCTFSPIMIGKGNFTSTTVTTTTSATATP
```

MscS/E.coli

```
MscS/E.coli   ............................................................
Msy1/S.pombe  .....RRHIGDYGVPDHVRVKLPWQQSANNVLITLFITSIMNLVEKVLMQLIAMSLHRRE
AN7571/MscA   .....NHHV......DGNSRTRDWENTLNKIIISVFVWTILNLIEKFLLQLIAMSFHRRT
AN6053/MscB   .....YNPRNKR...EGDTETKSWEKSVKNVLFAFLVCALIFLGEKTLVQLISISYHRKQ
Msy2/S.pombe  TASASSNAVESVFVTKTAASVPSWIKVITKILGAAVVTSIVLLLEKIFLHFIGFHYHEVQ
```

MscS/E.coli

```
MscS/E.coli   ............................................................
Msy1/S.pombe  YESRILYNKFAINELARLYGYARQRSFDFKDAIHRAQADVFKFA.....DHQHGKKRAAA
AN7571/MscA   YSDRIEINKFQIGSLTKLYAFSRNKIAETDEAFEEKQ.................DKSGSG
AN6053/MscB   FDARIKESKRNINLIGILYDASRSMFPMYCKEFREDDAIISDSILLGGPETGRPGHSRSN
Msy2/S.pombe  YQYRITDNKRNTAVLAKLLTAALDAPYHDSPRVRRQDYLLGLI......DTRSMSESKGS
```

MscS/E.coli

```
MscS/E.coli   ............................................................
Msy1/S.pombe  ARVAQNALNKTTYKAISAFNFATDMVNKVAGEITNREVEKSSSPKSVVLHLLKTTRGCQS
AN7571/MscA   AKTPLRYAGKARGLALGALNKVGDVAGAVAADFTGRKANSSSHPSQVVIALLRTTAGCQT
AN6053/MscB   SAAPLRFIRGVQQNVGRIGGKITGALGDVAHEITGKQVFNSSAARSIVSEALERRRSSEA
Msy2/S.pombe  GNGKLRKVKKISKNAKRIFSKTRNAISTAFTDMLGKHAKDLTPEQEFILETIRSKKKCLA
```

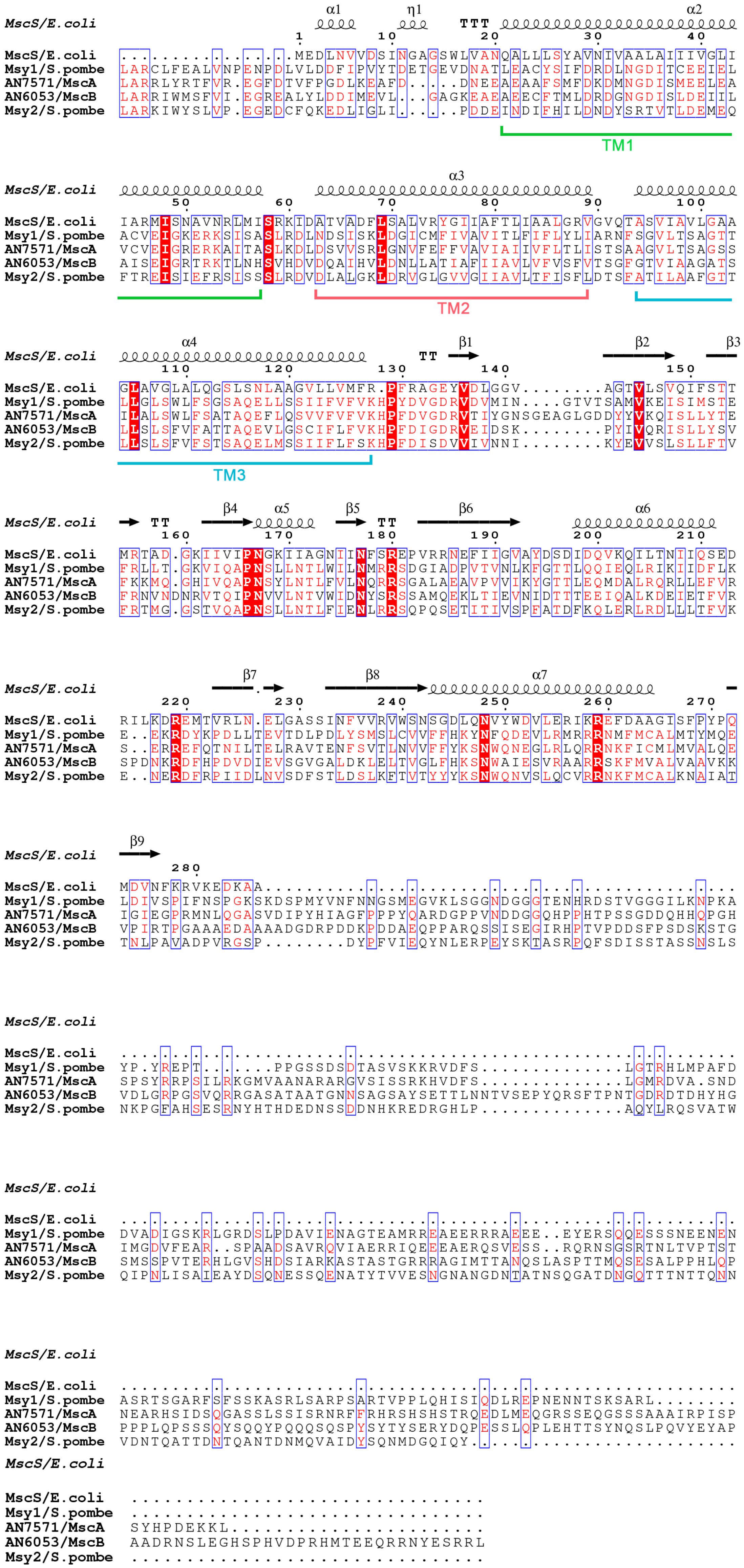

Figure S1. Multiple sequence alignment of MscA and MscB and representative MscS-like proteins. The alignment shows a similarity of MscA and MscB with MscS from *E. coli* and Msy1 and Msy2 from *S. pombe*. Topology predictions are also shown on top of each alignment and the 3 TM helices that correspond to MscS are indicated in green, red and blue colour (α2 = TM1, α3 = TM2, α4 = TM3). The figure was made using http://multalin.toulouse.inra.fr.

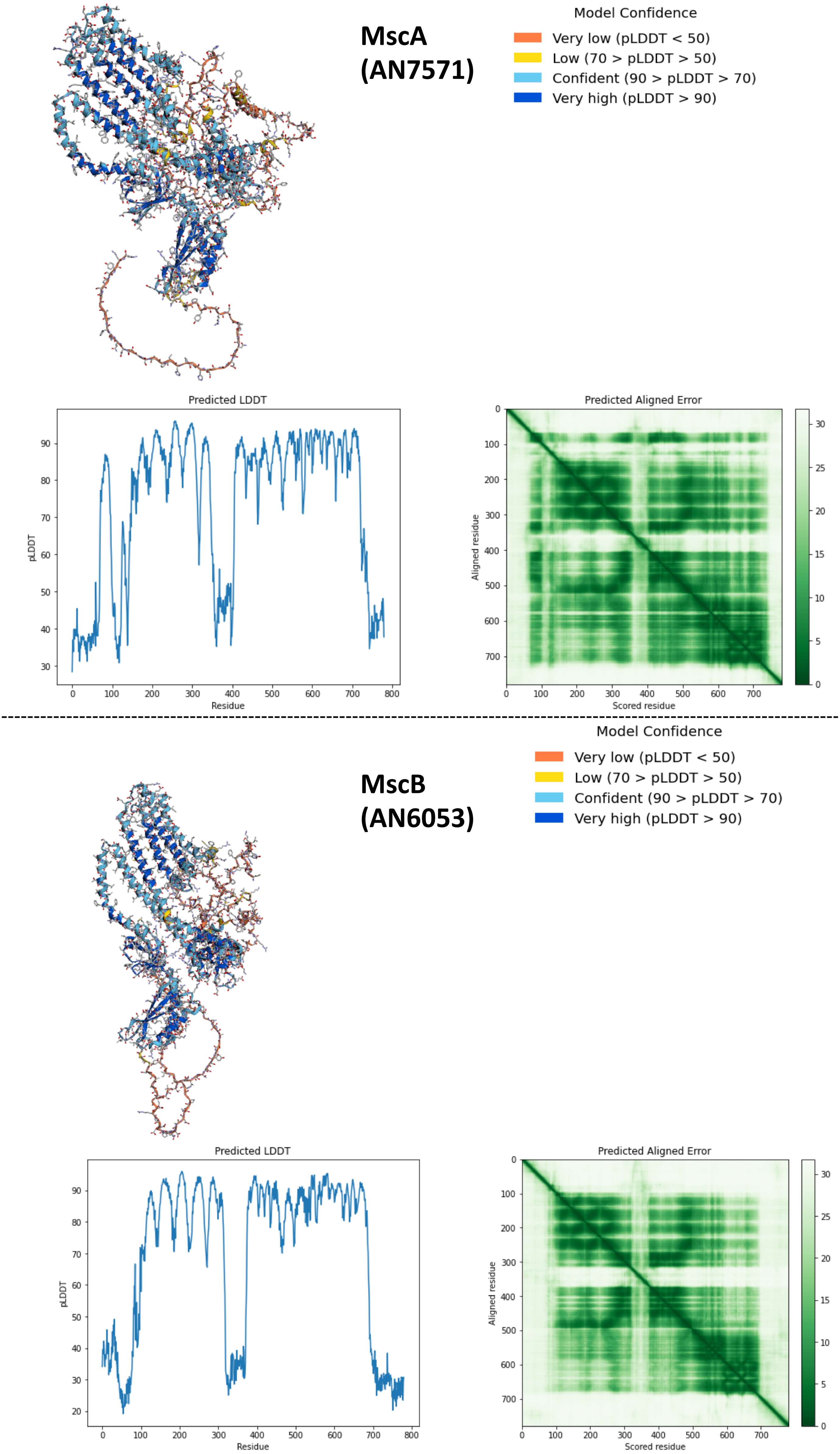

Figure S2 AlphaFold predicted model and confidence scores for MscA and MscB. Residues 1-780 of single subunit(s) MscA and MscB were used in analysis and prediction by Alphafold Colab.
